# A strengthening the reporting of observational studies in epidemiology (STROBE): Are HE4 and CA 125 suitable to detect a Paget disease of the vulva?

**DOI:** 10.1097/MD.0000000000024485

**Published:** 2021-02-05

**Authors:** Miriam Dellino, Giulio Gargano, Raffele Tinelli, Carmine Carriero, Carla Minoia, Skrypets Tetania, Erica Silvestris, Vera Loizzi, Angelo Paradiso, Porzia Casamassima, Antonio Tufaro, Gennaro Cormio, Vito Michele Garrisi

**Affiliations:** a Gynecologic Oncology Unit, IRCCS Istituto Tumori “Giovanni Paolo II” Bari; b Department of Obstetrics and Gynecology, “Valle d’Itria” Hospital, Martina Franca, Taranto; c Department Interdisciplinary Medicine, Unit of Obstetrics and Gynecology, University of Bari “Aldo Moro”; d Haematology Unit, IRCCS Istituto Tumori “Giovanni Paolo II” Bari; e Haematology Unit, National Cancer Center, IRCCS Istituto Tumori “Giovanni Paolo II”, viale O. Flacco 65, Bari, Italy; Clinical and Experimental Medicine PhD Program, University of Modena and Reggio Emilia; f Department of Biomedical Sciences and Human Oncology, Unit of Obstetrics and Gynaecology; g IRCCS Istituto Tumori “Giovanni Paolo II”; h Clinical Patology Laboratory, IRCCS Istituto Tumori “Giovanni Paolo II”; i Istitutional Biobank, IRCCS Istituto Tumori “Giovanni Paolo II” Bari, Italy.

**Keywords:** cancer antigen125, human epididymis protein 4, Paget disease of the vulva

## Abstract

Paget disease is a complex disorder that can be identified in the breast (mammary Paget disease) or in other locations (extramammary Paget's disease) such as ano-genital skin (Paget disease of the vulva -PVD). This condition is associated with low mortality, but a late diagnosis and recurrence can negatively impact the prognosis. Therefore, the main objective of this study is to evaluate if the human epididymis protein 4 (HE4) and cancer antigen125 (CA125) can promote recognition of PVD in early stages and during the relapses.

we have conducted a prospective, observational and laboratory-based study, that included 50 patients, whose 25 healthy women represented the control group and 25 PVD patients, which have been operated in our Oncology Institute, from May 2017 to September 2019. Both in the control group and in PVD patients, the CA-125 and HE4 were evaluated before surgery and after 6 months. Finally, a comparison of markers serum level, both between before/after surgery and with control group, and a ROC (Receiver Operating Characteristic) curve were performed.

Dosing the markers in PVD patients, 3/25 (12%) showed a higher value of CA125 and 11/25 (44%) an increased HE4. In addition, after surgical treatment there were no statistically significant difference between levels of CA-125 (*P* = .3) and HE4 (*P* = .19). On the other hand, comparing HE4 in PVD patients with the control group, a statistically significant difference was found (*P*-value = .0036). Contrary, comparing CA-125 in PVD patients with the control group (*P*-value= .1969), no statistically significant difference was evidenced. Moreover, ROC (Receiver Operating Characteristic) curve showed low sensitivity and specificity for CA125 with area under curve (AUC) = 0.5608. Instead, the ROC curve of HE4 revealed a sensitivity and specificity of 76% and 88% respectively (AUC = 0.7408) using a cut-off at 90 pmol/L.

Despite the limited cases, our data showed that CA125 is not a sensitive marker for PVD. On the other hand, in 44% of PVD we’ve seen an increase in HE4. So, this could be a starting point for further research that could confirm the possibility to use this marker in order to support PVD early identification.

## 1 Introduction

Human Epididymis Protein 4 (HE4) is a secreted glycoprotein, encoded by the omolologist gene^[1]^ and is part of the WFDC diprotein family (serum nucleo/four disulphide). HE4 is only slightly expressed at the epithelium level of respiratory and reproductive organs, while it is over-expressed in ovarian cancers.^[2]^ Therefore, it has been suggested as a complementary or alternative serum marker to carbohydrate antigen 125 (Carbohydrate Antigen 125 -CA125) for ovarian cancer (OC) risk assessment in presence of ovarian neoformation.^[3]^ Several studies demonstrate that HE4 has a sensitivity similar to CA125 and a greater specificity in patients with OC, while CA125 is reported in multiple physiological and pathological conditions such as pregnancy, menstruation, endometriosis.^[4]^ Whereas, the combination of CA-125 and HE4 in ovarian pathology, showed a sensitivity of 76.4% and a specificity of 95%.^[5]^ Despite, the undisputed validity of these markers in OC patients, the potential use in other types of cancers is still under investigation.^[6]^ In particular, our goal is to evaluate the variation of CA125 and HE4 in patients with diagnosed of Paget disease of the vulva (PVD). Paget disease represents a rare cancer (incidence of 1/100,000), most commonly found in postmenopausal women (Caucasian ethnicity), that can be located in the breast (mammary Paget disease)^[6]^ or anogenital area.

(Extramammary Paget's disease). Furthermore, 54% of PVD could be associated with lesions in other places (breast, intestine, bladder)^[7]^ and with the coexistence of invasion areas (adenocarcinoma) characterized by the presence of Paget cells infiltrating the underlying dermis.^[8]^ At the vulvoscopic examination, PVD is identified for the presence of a red and white eczematoid plaque, with a papillomatous and sometimes ulcerated surface^[9]^ and is often associated to irritation, itching, burning and vulva pain but without any pathognomonic symptoms.^[10]^ Consequently, PVD is often diagnosticated only in presence of very extensive disease with consequently indication to a demolition surgery and with a high percentage of local recurrence (30%–35%).^[11]^ Therefore, the aim of this study is to assess the effectiveness of CA-125, HE4 markers, to value the presence of PVD in women with a vulvar lesion. On the other hand, a secondary endpoint is to compare marker dosage in PVD patients, 6 months after surgery and in relation with group control.

## 2 Material and methods

This is a prospective, observational, laboratory-based study, concerning the dosage of biomarkers on peripheral blood of 50 consecutive patients submitted, of which 25 patients (average age at diagnosis of: 72.0 years, min–max: 50.0 to 82.0 years, Caucasians, menopause) with histological diagnosis of PVD and surgically treated by Gynecologic Oncology Unit in National Cancer Research Centre, Istituto Tumori “Giovanni Paolo II” of Bari between 2017 and 2019. Menopause status was defined as the absence of menstruation for more than 6 months, the presence of clinical signs of menopause, or with a history of hysterectomy. On the other hand, 25 healthy voluntary subjects, with similar characteristics (median age: 68.0 years, min–max: 60.0 to 91.0 years, Caucasians, in menopause), who usually perform the periodic follow-up (gynecological visit and ultrasound) for oncologic prevention, were enrolled. Consequently, in the healthy volunteers, a blood sampling for the dosage of CA-125 and HE4 was performed, after obtaining informed consent to participation in the study and to use personal data reported on specific database. Similarly, during a pre-inclusion visit of 25 patients with PVD, all demographic information, anamnestic, histological and diagnostic examination (pelvic examination, transvaginal ultrasound, Pap-test, chest X-ray, mammography, cystoscopy, and colonoscopy) have been collected and reported on specific database. All participants have been properly informed about research protocol, approved by the Institutional Review Board and subsequently they have also signed an informed consent both for participation of the study and for the use of personal data. In addition, on the day of surgery, during the preoperative evaluation, a blood sample was collected to perform CA125 and HE4 assays. In order to minimize variable effects due to sample collection, processing, and storage temperature, all blood samples were managed in the same manner without any protocol amendment during the entire collection period. The blood specimens were collected in serum separation tubes (Becton Dickinson, Franklin Lakes, NJ, USA), allowed to clot for thirty minutes, and then centrifuged at 3500 rpm for 15 minutes for the subsequent routinely use. The HE4 and CA125 levels were detected before and after surgery with an immune-enzymatic assay (Roche Diagnostics S.p.A.) according to manufacturer's guidelines. Concerning CA-125, the reference values are less than 35 U/mL,^[12]^ for HE4, the predetermined thresholds in menopausal patients’ status, are less than or equal to 140 pmol/L.^[13,14]^ Serum markers results and the histopathological analysis were received after surgery, by a gynecologic pathologist and the pathological diagnosis of PVD has been identified by 2 pathologists of our Institute and has been defined as PVD “Invasive” the extension for at least 1 mm beyond basal membrane while the “positive margin” has been defined in case of the presence of cells within 1 mm of the surgical margin. During clinical follow-up (6 months after surgery, range 3–9 months) the tumor markers dosage was repeated and the comparison of serum levels before/after-surgery and with control group was performed.

## 3 Results

**Figure 1:**
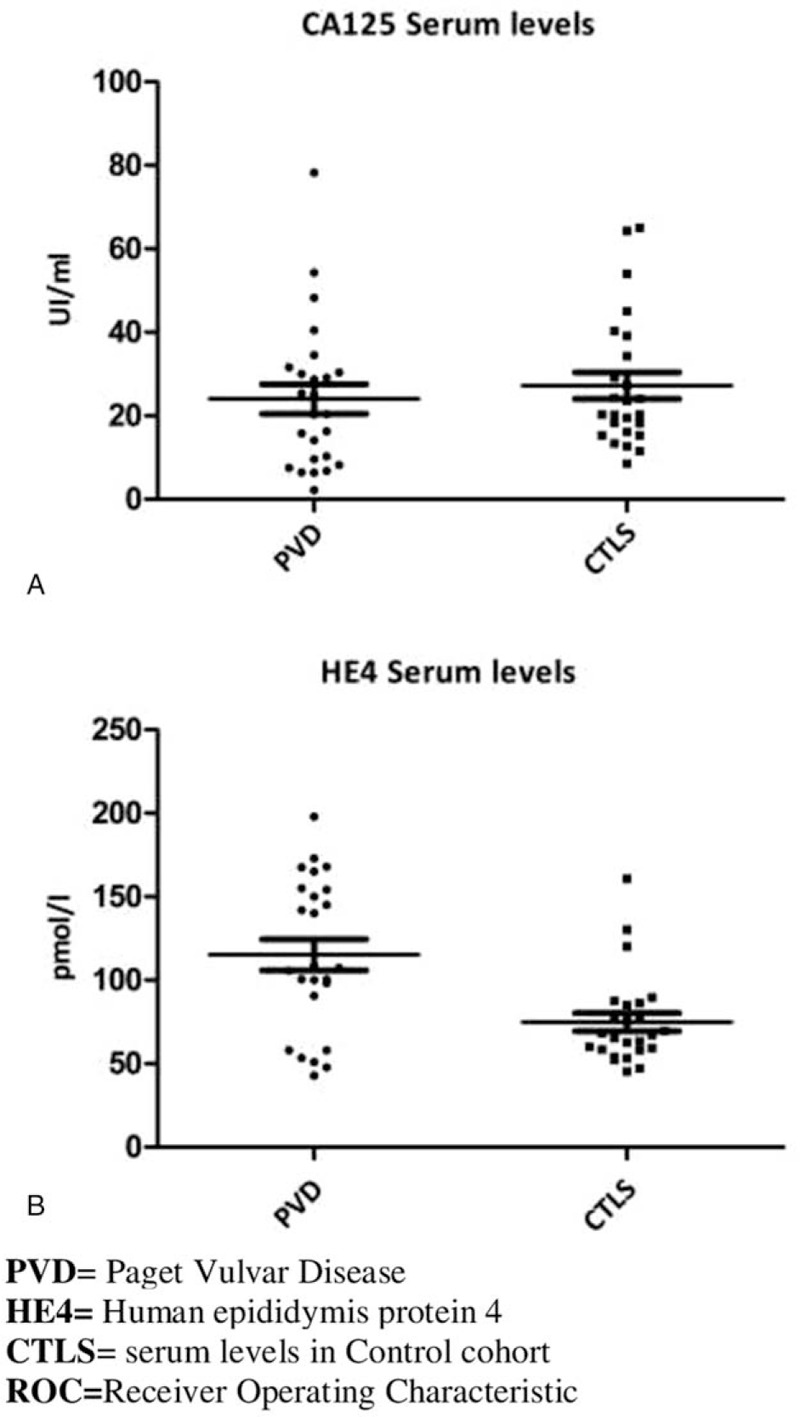
(A) The CA 125 serum level's comparison between PVD patients and control cohort individuals showed a lack of statistically significant differences (*P*-value >.005). (B) The HE4 serum level's comparison between PVD patients and control cohort individuals showed a statistically significant differences (*P*-value <.005).

**Figure 2:**
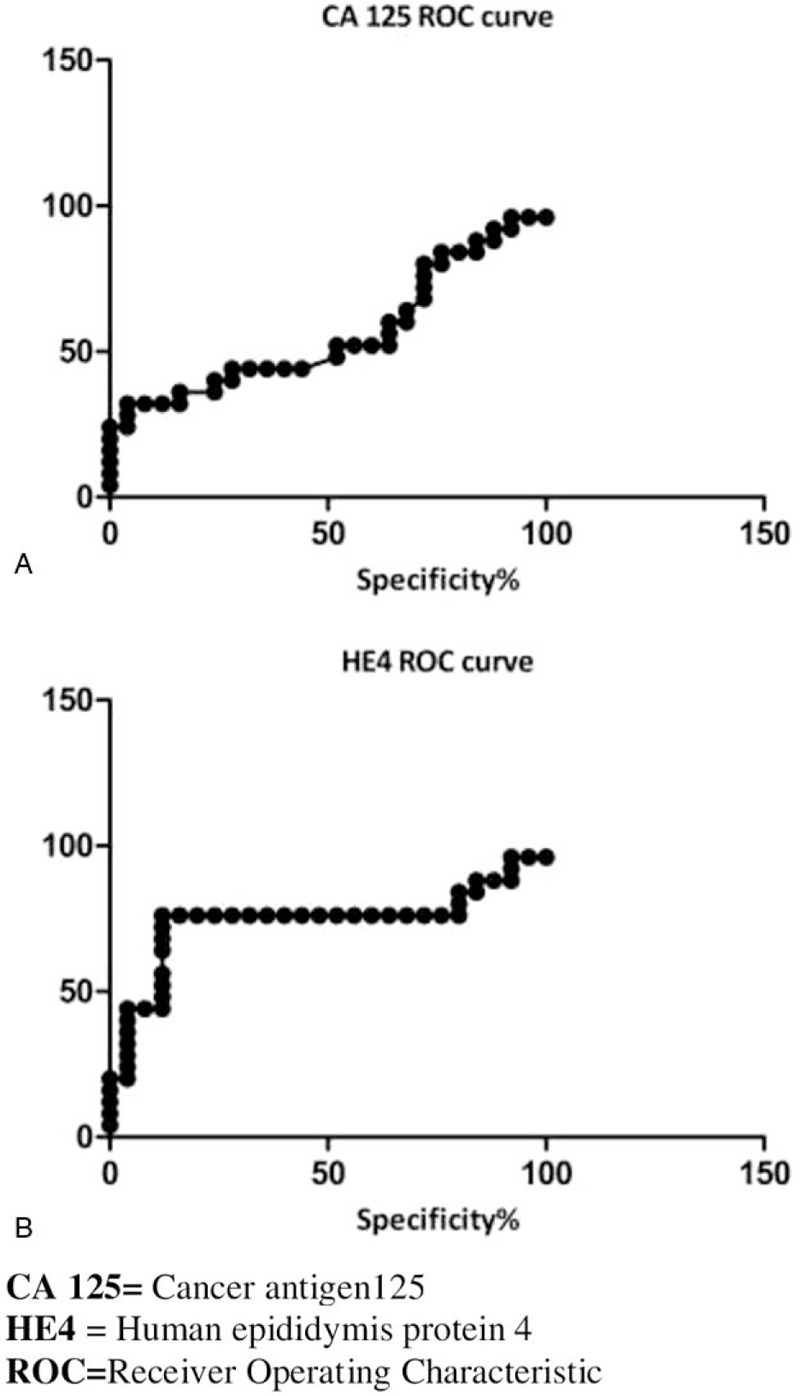
(A) ROC Curve analyses confirmed the uselessness of the CA 125 for PVD (sensitivity = 80%, specificity = 24%; AUC = 0.561). (B) In the HE4 ROC Curve analysis, using a cut-off of 90 pmol/L sensitivity was greatly improved (sensitivity = 76%, specificity= 88%; AUC = 0.741).

Fifty women were initially enrolled, from May 2017 to September 2019, whose 25 represented control group and 25 with PVD histological diagnosis. Among PVD patients, 3/25 (12%) presented a higher value of CA125 and 11/25 (44%) an increased HE4. In addition, was not recognized a statistically significant difference of CA-125 (*P* value = 0.3698) and HE4 (*P* value = 0.1969) after surgical treatment and also comparing CA-125 of PVD patients with the control group (*P* value = 0.1969 Fig. 1 A). On the other hand, comparing HE4 in PVD cases with the control group, a statistically significant difference was found (*P*-value = 0.0036 Fig. 1B). Moreover, in the control group, 6/25 patients (24%) had CA-125 levels above the cut-off, 1/25 (4%) had HE4 levels higher than cut-off. No statistically significant differences were observed comparing before and after CA 125 and HE4 values (data not shown). ROC Curve showed low sensitivity and specificity for CA125 (AUC = 0.561) while the ROC curve of HE4 revealed a sensitivity and specificity of 76% and 88% respectively (AUC = 0.7408) using a cut-off at 90 pmol/L (Fig. 2A and B). Furthermore, we have observed that 10/25 (40%) of PVD patients were completely asymptomatic, on the contrary, 9/25 (36%) PVD patients reported specific symptoms (itching, burning, and vulva pain) with a duration of 28.6 months (interval 12–40 months) before diagnosis. Furthermore, 2/25 (8%) patients performed local medical treatment (respectively imiquimod and fluorouracil) before surgery, without any benefit. All patients underwent surgery, including 4/25 (16%) local excision, 8/25 (32%) simple vulvectomy, 12/25 (48%) extended vulvectomy. On the pathological examination, 2/25 (8%) patients presented an invasive disease so a lymph-adenectomy was performed and a single inguinal lymph node involved was reported. Moreover, in 8/25 patients (32%) surgical reconstruction was necessary, but no patient needed of a blood transfusion during or after surgery. Finally, no patient has received adjuvant treatment with radiotherapy, after primary surgery and the status of margins was available for all patients, of which 11/25 (44%) had positive margins without any relationship with the extent of surgery.

### 3.1 Statistical analysis

In order to compare CA125 and HE4, before and after surgery and with control group, the Kruskal-Wallis while t-test was used. The level of statistical significance has been set to *P*-value < .005. ROC (Receiver Operating Characteristic) curve and relative AUC (Area under curve) were calculated both for CA125 and HE4. Statistical analyses were performed using Graph pad Prism 5.0 software.

## 4 Discussion

PVD could be diagnosed after a vulvoscopic examination, which is usually performed through a colposcopic or a dermatoscopic inspection.^[15]^ On the other hand, the applying of specific reactive (acetic acid and Lugol's iodine), commonly adopted in cervical cancer screening, is not indicated for vulvar lesions evaluation. Consequently, the use of non-invasive procedure as markers serological dosage (HE4 and CA125) to support the diagnosis could be extremely helpful in order to guide the early PVD diagnostic-therapeutic process and the identification of recurrences. This is especially proper, regarding a rare disease as PVD, whose clinical knowledge are limited and the clinical interpretation may be equivocal.^[16–18]^ Indeed, the differential diagnosis includes skin candidiasis, seborroic dermatitis, psoriasis, Bowen disease and melanoma.^[19]^ Therefore, since an exceptional number of PVD cases have come to the observation of our clinic and some of these had an increase in HE4, that is expressed also in epithelial tissues, we tried to establish if this recently proposed biomarker could be associated with the presence of PVD and consequently suitable in PVD diagnosis and/or management. Our data shows that, using the assessed HE4 cut-off (140 pmol/L), 44% of patients with PVD have a higher HE4 value and compared with HE4 dosing in the control group a statistical difference was found. Consequently, this marker could direct the clinician to perform a vulvar biopsy in case of suspected lesion and during the follow-up. In contrast, CA125 evaluation, seems to be not indicated in the presence of PVD. This assessment is further confirmed by the absence of a significant difference of CA125 both after surgery and compared to the control group. Moreover, the ROC analyses of HE4 highlighted some suggestion to be discussed. In particular, by lowering the cut-off threshold from 140 pmol/L to 90 pmol/L, the sensitivity improved greatly from 44% to 76% with an acceptable specificity of 88%.

Nerveless, concerning PVD and oncological markers, particularly HE4, no data are presented in literature, so it is difficult to compare our result. On the other hand, a recent study reports the assessment of tumor markers in vulvar cancer, showing that the best diagnostic performance was achieved for Carcinoembryonic Antigen (CEA).^[20]^ Indeed, a significantly higher values of CEA in affected patients compared to control groups was found. Nevertheless, even in the latter case, it is far from establishing the real utility of this biomarker and the potential introduction in clinical practice.

## 5 Conclusions

PVD can remain undiagnosed for several years, so frequently it is recognized as an extensive vulvar lesion which needs the use of demolition surgery and subsequent plastic-reconstruction.^[21]^

Therefore, the search for serological markers to assist the early detection of PVD, would allow the identification of limited and non-invasive forms and the use of alternative approaches such as imiquimod and photodynamic treatment (currently off label).^[22]^

Actually, none of the markers analyzed are helpful in the specific identification of PVD, but the increase HE4 value, in vulvar lesion, could support clinician decision to perform a biopsy and early detection of PVD that consequently could improve the mortality and morbidity.^[23]^

It is also necessary to consider limitations of this study, because of restricted number of cases and for the data absence in the available publications concerning the association between PVD and serological marker. Therefore, this experience could be a valid tool to be used in routine clinical practice and possibly, a cornerstone for further discussion on the topic also considering the rarity of this pathology. It also may provide useful recommendations for national and international gynecological society.

## Acknowledgment

This research project has been supported in part by the Apulian Regional Project “Medicina di precisione”.

## Author contributions

**Data curation:** Vera Loizzi.

**Formal analysis:** Carla Minoia, Gennaro Cormio.

**Funding acquisition:** Carla Minoia, Angelo Paradiso.

**Investigation:** Erica Silvestris.

**Methodology:** Porzia Casamassima, Vito Michele Garrisi.

**Project administration:** Antonio Tufaro.

**Resources:** Skrypets Tetania.

**Supervision:** Giulio Gargano, Carmine Carriero, Angelo Paradiso, Gennaro Cormio.

**Validation:** Gennaro Cormio.

**Visualization:** Vera Loizzi, Gennaro Cormio.

**Writing – original draft:** Miriam Dellino, Vito Michele Garrisi.

**Writing – review & editing:** Raffele Tinelli.
